# Identification of HCV Resistant Variants against Direct Acting Antivirals in Plasma and Liver of Treatment Naïve Patients

**DOI:** 10.1038/s41598-017-04931-y

**Published:** 2017-07-05

**Authors:** V. Stalin Raj, Gadissa Bedada Hundie, Anita C. Schürch, Saskia L. Smits, Suzan D. Pas, Sophie Le Pogam, Harry L. A. Janssen, Rob J. de Knegt, Albert D. M. E. Osterhaus, Isabel Najera, Charles A. Boucher, Bart L. Haagmans

**Affiliations:** 1 Department of Viroscience, Erasmus Medical Center, Rotterdam, The Netherlands; 2 Viroclinics Biosciences BV, Rotterdam, The Netherlands; 3 Virology Discovery, Pharma Research Early Development Hoffmann La Roche, Nutley, NJ USA; 4 Department of Gastroenterology and Hepatology, Erasmus Medical Center, Rotterdam, The Netherlands; 5 Division of Gastroenterology, University Health Network, Toronto, Canada; 6 Artemis One health, Utrecht, The Netherlands; 7 Center for Infection Medicine and Zoonoses Research, University of Veterinary Medicine, Hannover, Germany

## Abstract

Current standard-of-care treatment of chronically infected hepatitis C virus (HCV) patients involves direct-acting antivirals (DAA). However, concerns exist regarding the emergence of drug -resistant variants and subsequent treatment failure. In this study, we investigate potential natural drug-resistance mutations in the NS5B gene of HCV genotype 1b from treatment-naïve patients. Population-based sequencing and 454 deep sequencing of NS5B gene were performed on plasma and liver samples obtained from 18 treatment- naïve patients. The quasispecies distribution in plasma and liver samples showed a remarkable overlap in each patient. Although unique sequences in plasma or liver were observed, in the majority of cases the most dominant sequences were shown to be identical in both compartments. Neither in plasma nor in the liver codon changes were detected at position 282 that cause resistance to nucleos(t)ide analogues. However, in 10 patients the V321I change conferring resistance to nucleos(t)ide NS5B polymerase inhibitors and in 16 patients the C316N/Y/H non-nucleoside inhibitors were found mainly in liver samples. In conclusion, 454-deep sequencing of liver and plasma compartments in treatment naïve patients provides insight into viral quasispecies and the pre-existence of some drug-resistant variants in the liver, which are not necessarily present in plasma.

## Introduction

Hepatitis C virus (HCV) is a positive-strand enveloped RNA virus, classified in the genus *Hepacivirus*, family *Flaviviridae*. This virus displays very high genetic variability, a primary problem for the development of an effective HCV vaccine and an explanation for the emergence of resistance during antiviral therapy. Accumulation of nucleotide substitutions in the virus has resulted in diversification into numerous subtypes and distinct genotypes^1, 2^. The genetic diversity is due to an error-prone RNA-dependent RNA polymerase, which generates on average 1.7 × 10^−3^ base substitutions per site per year, a high virion production rate (up to 1 × 10^12^ particles produced per day), recombination, and deletion^3–5^. Consequentially, mutations including those associated with drug resistance are spontaneously generated many times daily in each patient and drug-resistant variants, therefore, may already pre-exist as a minor population within a pool of closely related virus variants called quasi-species^6, 7^.

Until recently, the only treatment for patients with chronic hepatitis C was a combination of pegylated interferon alpha (Peg-IFN) and ribavirin (RBV), shown to be relatively ineffective with a viral eradication rate of approximately only 50%^8^. Besides, this antiviral therapy is associated with numerous side effects, which excluded up to 50% of patients upfront from antiviral therapy^9^. There is a clear medical need for more efficacious therapies, and nowadays, several novel direct-acting antivirals (DAAs) that target NS3/NS4A protease, NS5B polymerase and NS5A protein either combined with Peg-IFN/RBV or INF-free combinations of DAAs have shown potent antiviral effects resulting in high cure rates in HCV- infected patients^10–12^. Antiviral therapy suppressing the wild-type virus but not the pre-existing resistant minority viruses, in due process, may function as a positive selective pressure leading to the rapid outgrowth of drug-resistant variants^13–17^.

The most common method of detecting drug-resistant variants in HCV-infected patients is population-based Sanger sequencing. Using standard Sanger sequencing methods, the abundant HCV diversity in chronically HCV-infected patients cannot be fully mapped as relevant proportions of minority variants can be missed^12^. Deep sequencing (DPS), however, now allows for identification of rare minority drug-resistant human immunodeficiency virus variants which are not detectable by standard sequencing techniques^18, 19^, and recent studies also identified minor drug-resistant variants in plasma of HCV-infected patients^12, 14–17^. In this study, we developed a DPS approach to obtain insight into HCV NS5B viral quasi-species and the presence of drug-resistance associated NS5B variants in the plasma and liver tissue of treatment naïve chronic HCV infected patients.

## Results

### Validation of the deep sequencing assay

To allow analysis of HCV quasi-species in liver and plasma of HCV-infected treatment-naïve patients and in-depth analysis of the presence of drug-resistant HCV variants, a DPS approach was developed to analyze a 339 bp nucleotide genome fragment spanning amino acid positions 226 to 337 of the NS5B region. The frequency and nature of potential errors were analyzed by comparing DPS sequence reads of an HCV-1b NS5B plasmid to a consensus sequence obtained from the same construct by Sanger sequencing. To examine errors introduced by PCR and DPS, the plasmid was quantified and diluted to 10^6^ copies per ml, amplified by conventional PCR, and sequenced using the deep sequencing protocol. The contribution of reverse transcription to the error rate of the protocol was analysed using RNA synthesized from the plasmid, after which cDNA, PCR, and 454-sequencing were performed.

**Figure 1:**
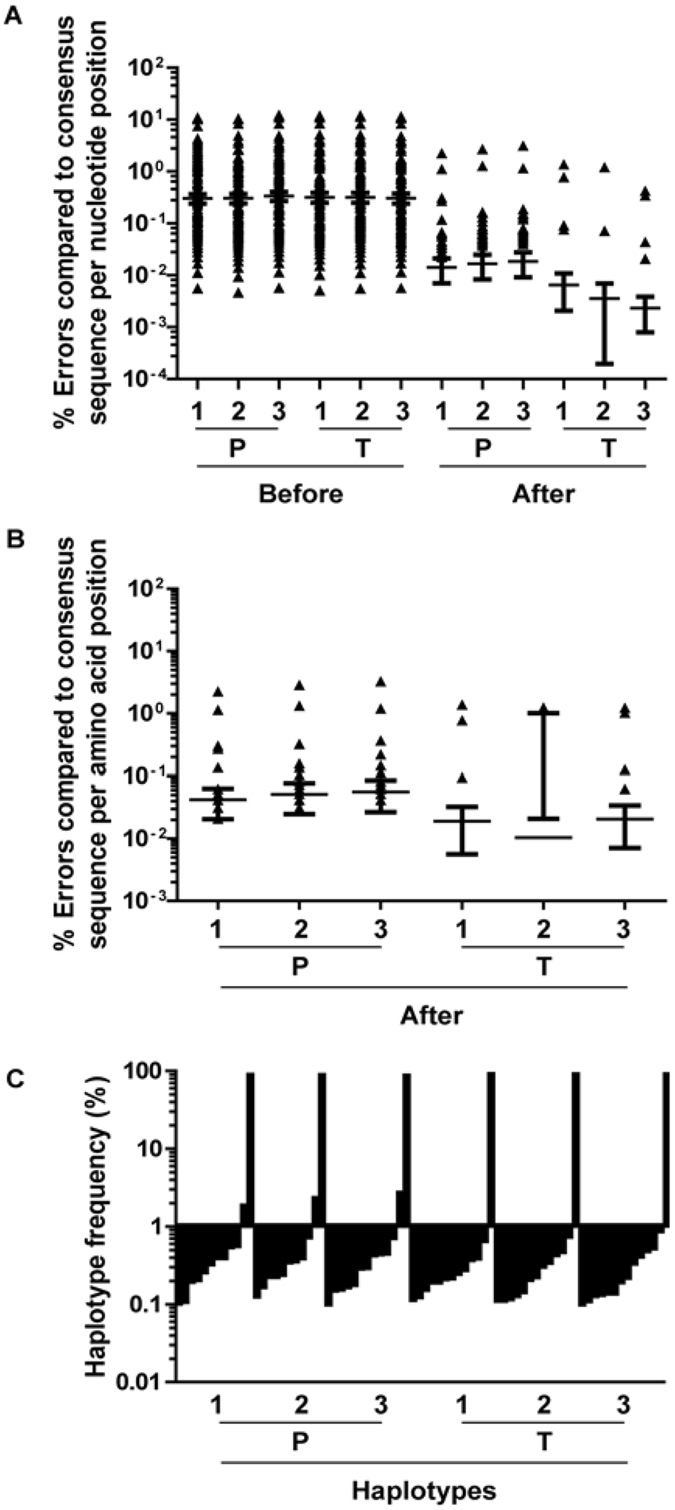
Validation of the next-generation sequencing assay. (**A**) A position-specific error rate per nucleotide was determined before or after read-cleaning algorithms were applied for the control experiments with plasmid (P) or RNA transcript (T) in 3 independent next-generation sequencing experiments (1–3) each. Mean ± sem are shown. (**B**) For confirmation, a position-specific error rate per amino acid position was determined after read-cleaning algorithms were applied for the control experiments with plasmid (P) or RNA transcript (T) in 3 independent next-generation sequencing experiments (1–3) each. Mean ± sem are shown. (**C**) Haplotypes and their frequencies in the dataset after read-cleaning were determined for the control experiments with plasmid (P) or RNA transcript (T) in 3 independent next-generation sequencing experiments (1–3) and plotted against each other with a cut-off value of 0.1%.

The raw sequence data generated in these experiments contained many errors, not uniformly distributed over the amplified region (Fig. 1A). Especially GC-rich areas in the sequence (8 to 11 bp GC-rich stretches) led to an increased number of insertions and deletions. A position-specific error rate per nucleotide was determined before read-cleaning algorithms were applied for the control experiments (Fig. 1A). The average error rate before read cleaning in all six experiments across the amplicon was similar and ranged from 0.3–0.34%, with on average 1.02–1.16 errors per read. The average error rate after read cleaning in all six experiments across the analyzed amplicon was more dissimilar although not significantly different, ranging from 0.014–0.018% for plasmids and 0.0023–0.0065% for transcripts (Fig. 1A). On average, every read retained 0.04–0.06 (plasmids) and 0.008–0.02 (transcripts) errors. These data indicate that most sequencing errors were introduced by PCR and DPS and not by reverse transcription. Similarly, position-specific error rates per amino acid were determined for translated amino acid sequences from the cleaned reads in the six control experiments (Fig. 1B). The average error rate after read cleaning was 0.042–0.056% errors per amino acid for plasmids (0.14–0.19 errors per translated read on average) and 0.010–0.021% for transcripts (0.03–0.1 errors per translated read on average) (Fig. 1B). This suggests that error introduction through the deep sequencing protocol is less pronounced when starting from RNA transcripts compared to plasmid DNA, although differences did not reach statistical significance.

The read cleaning approach aims to purge errors potentially caused by DPS (but not by PCR or reverse transcription). To examine the effectiveness of the deep sequencing analysis, two sets of reads with different 454-specific error profiles were artificially simulated from the sequence of the plasmid. Before read cleaning, the average error rate of the simulated reads was 0.77% and 3.2%, respectively, thereby exceeding the number of errors effectively encountered in the six control experiments. After analysis, all errors were removed (average error rate of ~0% at every position, not shown), indicating the effectiveness of the read cleaning approach in the removal of errors that are typically associated with the deep sequencing technique.

For haplotype reconstruction, the set of reads after read cleaning was translated into amino acid sequences and analyzed for redundancy (Fig. 1C). One dominant haplotype was encountered in these control experiments, with a frequency of ~90%. Multiple minor haplotypes that are generated by remaining sequencing errors, which together add up to ~10%, are still present. Most of these errors occur in less than 0.1% of the reads but at certain amino acid positions especially in the control experiments starting from the plasmid DNA, a haplotype generated by sequencing error can occur in percentages as high as ~2.5% of the total cleaned read population (Fig. 1C).

As the error rates per nucleotide position are highly diverse, it is unreliable to define a cut-off value based on average error rate per nucleotide position. Instead, each nucleotide or amino acid position should be evaluated separately, which can be achieved for specific positions of special interest, for example, known drug resistance positions. If the variation across the entire genome is of interest, another type of analysis with reconstruction of haplotypes can be performed and the cut-off value can be determined based on data from control experiments with plasmid DNA or RNA transcripts, which in our case was placed at a haplotype threshold frequency of 1% (Fig. 1C).

### Analysis of HCV quasispecies in liver and plasma of chronically HCV-infected patients

**Figure 2:**
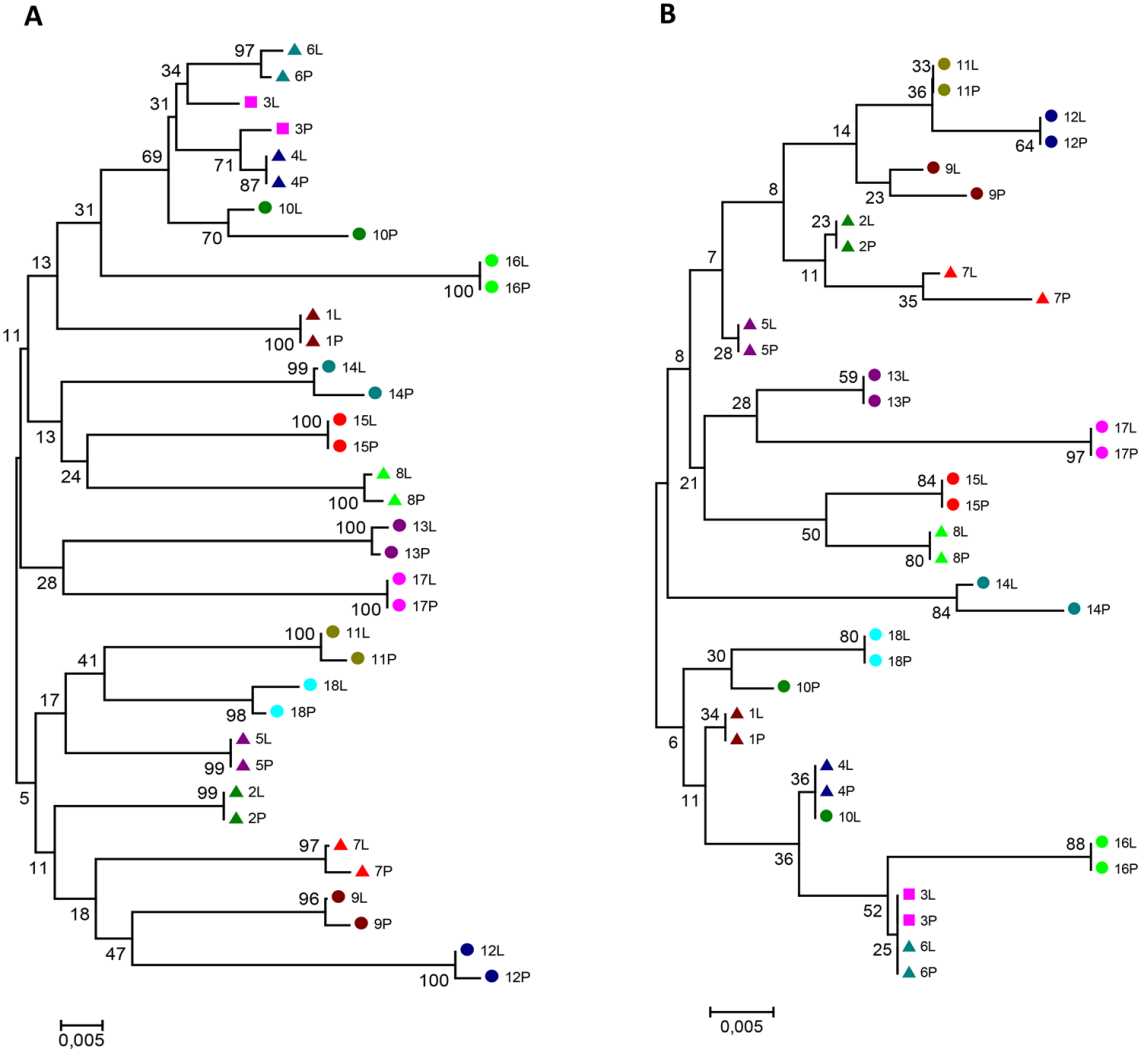
Phylogenetic analysis of the partial NS5B consensus nucleotide (**A**) and deduced amino acid (**B**) sequences from plasma and corresponding liver biopsies from HCV patients. Phylogenetic trees were generated using MEGA5, with the neighbour-joining method with p-distance model and 1,000 bootstrap replicates. Bootstrap values are shown. The different patients are indicated by colour, shape, and numbering with liver (L) and plasma (P) sequences indicated.

The HCV quasispecies in liver tissue and plasma from eighteen chronically infected, untreated individuals infected with HCV-1b were analyzed using DPS. All samples were successfully amplified by the NS5B nested-PCR method. The number of RNA molecules subjected to cDNA synthesis was not significantly different between plasma and liver samples (data not shown). Phylogenetic analysis of either nucleotide or amino acid consensus sequences determined by Sanger sequencing revealed that plasma and corresponding liver sequences clustered together (Fig. 2). After DPS, we obtained a total of 862,618 reads from 18 paired liver tissue and plasma samples with a median number of reads per sample of ~24,000 (range 8,605 to 37,164, Supplementary Table S3). Subsequently, the data were cleaned and corrected for DPS sequencing errors to obtain relatively error-free reads. During this process, reads were discarded from each sample and a median of ~23,000 (8,400 to 36,292) reads remained for each sample.

**Figure 3:**
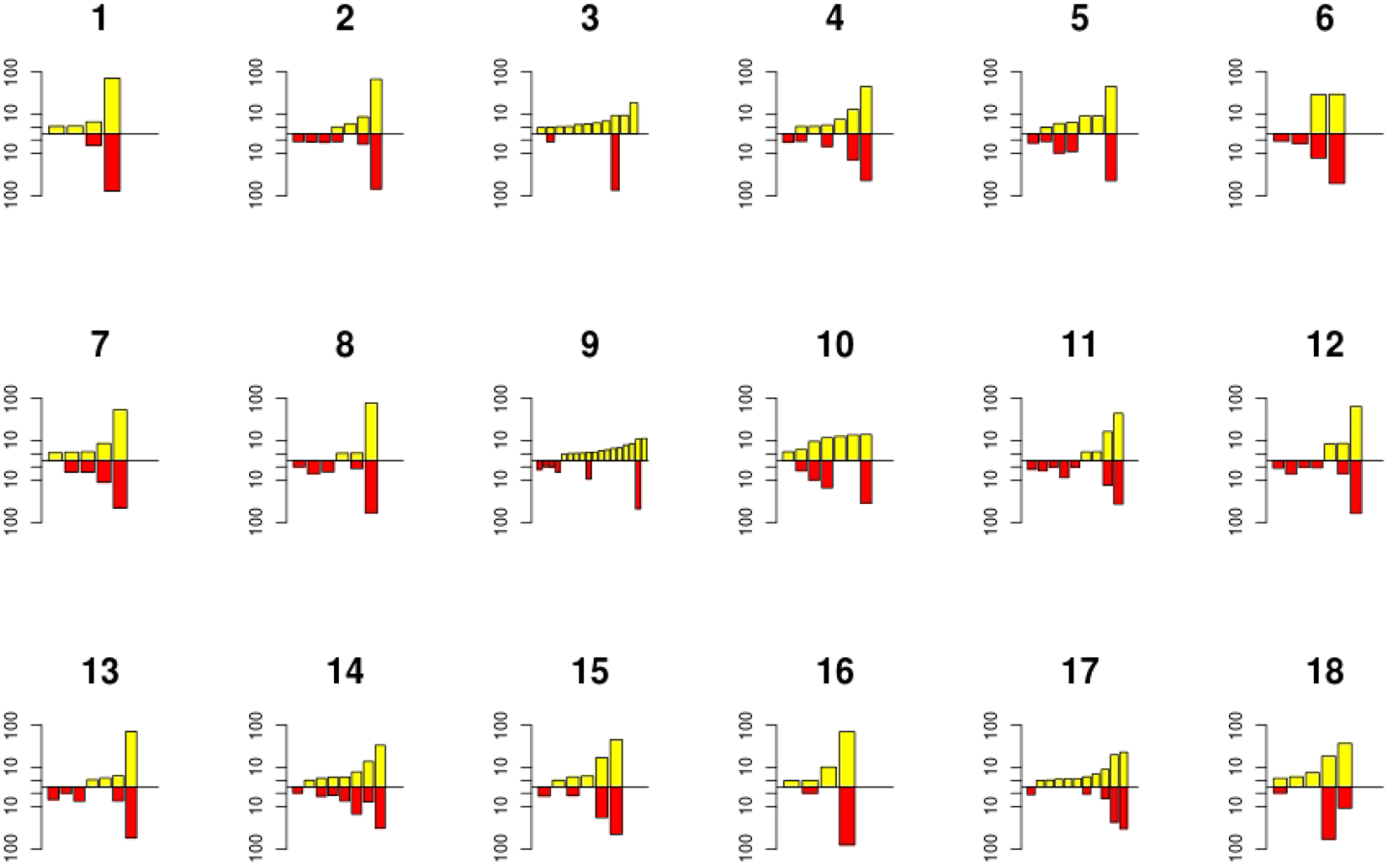
The number and variation of HCV haplotypes in 18 paired liver and plasma samples. The graphs indicate the number and frequency of each haplotype with a cut-off of a frequency of 1% or higher per haplotype, per liver (yellow) and plasma (red) sample of each patient (indicated by a number above the graphs).

For haplotype reconstruction, the set of corrected reads after read cleaning was translated into amino acid sequences and analyzed for redundancy. The frequency of the haplotypes was determined by counting the number of amino acid sequences with an identical sequence. Most haplotypes in each sample were found at very low frequencies (less than 0.1%). Based on our control experiments with plasmid DNA and RNA transcripts, these haplotypes could be generated through sequencing errors, not cleaned from the reads. To achieve comparability between the different samples while maintaining a concise number of haplotypes, only haplotypes occurring at a frequency of 1% or more were taken into account for further phylogenetic and population analyses (Fig. 1C). On average, 5.8 (range 2 to 14) and 4.6 protein haplotypes (range 2 to 7) were observed per liver or plasma sample, respectively. These haplotypes represented 79.3% of the whole population of sampled reads (Supplementary Table S3). Each sample consisted of one or two major protein haplotypes and several minor protein haplotypes (Fig. 3). In almost all patients, the most prevalent haplotype in plasma was also present in the liver (Fig. 3), except for patient 3, consistent with the results presented in Fig. 2A. Compartment unique sequences were observed in all patients (Fig. 3). The Simpson’s diversity index of the haplotype population was estimated, considering the number of haplotypes, the total number of sequences, and the proportion of the total number of reads found for each haplotype. The diversity index in both compartments was found to be comparable for most patients analyzed (Supplementary Table S3).

### Variability at drug resistance positions

To determine whether NS5B gene drug-resistant variants pre-existed, specific codon positions implicated in resistance to NS5B inhibitors or associated with response to IFN/ribavirin treatment were analyzed. To note that within the region analyzed, codon positions 282, 316, and 321 have been implicated in resistance to NS5B inhibitors^11, 20–22^; the S282T mutation, for example, confers resistance to 2′-modified nucleotide analogues including the recently approved sofosbuvir.

**Figure 4:**
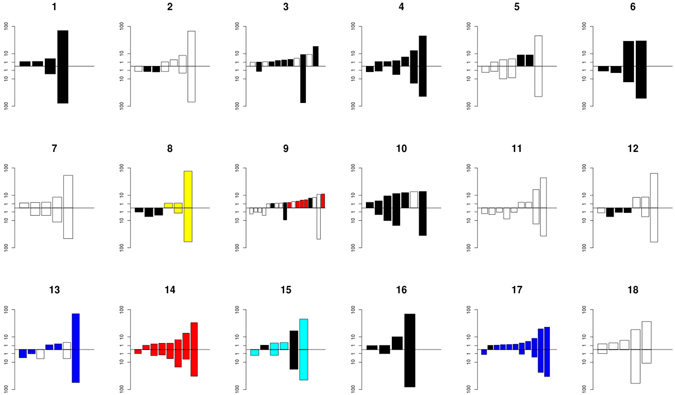
The number and variation of HCV haplotypes in 18 paired liver and plasma samples. The graphs indicate the number and frequency of each haplotype with a resistance mutation. The upper bars indicate the liver samples, whereas the lower bars represent plasma samples from the same patient (indicated by a number above the graphs). The colours represent different variants: Black, C316N; Red, C316H+V321I; Blue, Q309R; Cyan, C316N+Q309R; Yellow, C316Y+Q309R. Only haplotypes with a cut-off of a frequency of 1% or higher per haplotype are depicted.

In our patient cohort, no codon changes from S282 (cut-off position-specific error rate 0) were detected either in plasma or in the liver by either Sanger or deep sequencing (Supplementary Table S4). Sanger sequencing did detect C316N, C316Y, and/or C316H mutations in 9 patient liver and/or plasma samples (50%), whereas deep sequencing detected such a mutation as minor variant in an additional 7 patients in liver and/or plasma samples (89%) with a cut-off of 0.2% based on the position-specific error rate determined in the control experiments (Fig. 4 and Supplementary Table S4). The V321I substitution was detected by Sanger sequencing in 1 patient (5.6%) and deep sequencing detected this mutation as a minor variant in 10 patients (55.6%) with a cut-off of 0 based on the position-specific error rate determined in the control experiments (Supplementary Table S4). Of interest, double mutations of C316H and V321I were observed in 2 patients in liver and/or plasma samples.

We analyzed six additional codons (310, 329, 244, 309, 326 and 333) that have been associated with a decreased response to IFN/ribavirin therapy (Supplementary Table S4), although none of these variants have been confirmed to be associated with virologic treatment outcome and nothing is known about potential functional associations with the mechanism of action of interferon and/or ribavirin refs 23–25. All codon positions showed a position-specific error rate in the control experiment of 0, except for position 333 for which the specific error rate was 0.4%. The A333E mutation was not encountered in any of the patients. The D310N mutation was found in 9 liver and 8 plasma samples as a minor variant in a total of 11 patients. The D244N mutation was found in 5 liver and 9 plasma samples as a minor variant in a total of 10 patients, whereas the S326G mutation was found in 8 liver and 9 plasma samples as a minor variant in a total of 12 patients. The T329I mutation was observed in 11 liver and plasma samples from 11 patients and the Q309R mutation was found in 14 liver and plasma samples as a minor variant and in 4 patients as a major variant. The Q309R mutation was found in conjunction with C316 mutations in three patients. Overall, 22/198 variants were detected in the liver but not in the plasma whereas less unique variants were observed in the plasma (12/198).

## Discussion

In this study, a deep sequencing approach was developed and validated to analyze liver and plasma HCV NS5B quasi-species and drug resistance-associated variants from eighteen treatment naïve patients. We optimised DPS protocols, data cleaning, and error correction strategies using a reference plasmid as input. The data indicated that the intra-assay precision was very high and that most sequencing errors were introduced by PCR and deep sequencing and not by reverse transcription. Although the average error rate after read cleaning was very low in the control experiments, ~0.01% and ~0.03% on the nucleotide and amino acid level, respectively, the range of the error rates per nucleotide or amino acid was 0–3%. Similar observations in control experiments with a similar deep sequencing platform for HIV-1 quasi-species analysis were obtained previously^18, 19^. Thus, each nucleotide- or amino acid-specific position should be evaluated separately, which can be achieved for specific positions of special interest, for example, known drug resistance positions. For the analysis of NS5B quasi-species in liver and plasma, haplotypes were reconstructed and based on the control experiments, a conservative cut-off was placed at a haplotype frequency of 1%. Interpretation of the data generated in this study requires some caution as the liver needle biopsy specimen may not be representative for an entire liver.

HCV quasi-species diversity has been implicated to play a role in HCV clearance and disease progression with a limited diversity being favourable for HCV clearance but not for disease progression^26–29^. In the majority of patients, the most prevalent haplotype(s) were identical in plasma and liver but compartment unique sequences were observed as reported previously^6, 13, 30–34^. Comparison of the Simpson’s diversity index showed that the extent of diversity was relatively similar between the liver and plasma compartments per patient in 15 patients. A deterministic evolution selecting for the fittest (dominant) strain can be envisaged, based on the apparent presence of the same dominant haplotypes in both plasma and liver in most patients. Although the absence of a unique minority haplotype from plasma does not imply the absence of that haplotype in the liver and vice versa, quite a number of compartment unique haplotypes were obtained. Two hypotheses may explain these observations. One possible explanation could be that extrahepatic HCV replication occurs or a constant and dynamic flow of viral minority quasi-species between the two compartments, plasma and liver, may not occur. Alternatively, minority haplotypes are being generated in some liver areas, but their fitness constraints apparently do not allow them to occupy a large part of the sequence space and therefore are not detected in plasma.

A new era in HCV therapeutics has arrived with the development of direct-acting antivirals therapy and the management of antiviral resistance. We determined whether pre-existing direct-acting antiviral drug resistance mutations in NS5B in plasma and liver tissue of treatment naïve chronic HCV infected patients occurred. Already quite a number of drug resistance mutations have been described using *in vitro* selection protocols or *in vivo*, among which the S282T, C316N,H, Y, and V321Y mutations confer resistance to NS5B (non)-nucleoside inhibitors^20–22, 35^. The NS5B S282T variant is associated with a decrease in replicative fitness *in vitro* and has hardly been yet encountered in clinical trials in patients^20, 22, 36–38^. For instance, in a recent comprehensive analysis of 1,344 HCV isolates focussing on the NS5B gene, S282T was present in just one isolate for each genotype 1a, 1b, 3, and 4 at frequencies of 0.17%, 0.24%, 1.24%, and 1.63%, respectively^39^. In our cohort of treatment-naïve patients, the S282T mutation was not detected not even as a minority variant either in plasma or liver tissue. However, the other nucleos(t)ide inhibitors resistant variant V321A was detected in 10 out of 18 patients (~56%) mainly as minority haplotypes. As non-nucleoside inhibitors bind more distantly to the active site of NS5B, resistance-associated variants often occur more frequently with these compounds^40^. Mutations that confer resistance to non-nucleoside inhibitors at position 316 in NS5B *in vivo* have been described in treatment-naïve patients at frequencies of 0.19–24% by Sanger sequencing analyses^24, 41–43^. We noticed this mutation in 16 out of 18 patients (~89%) either as dominant (50%) or minority haplotypes (39%). These values are much higher than those obtained with Sanger sequencing and suggest that the presence of certain drug-resistant variants prior to treatment and minority variants are relatively high. In addition, variants containing two mutations in the same genomic strand involved in drug resistance against different compounds were encountered. Two patients showed mutations described in conferring resistance to non-nucleoside compound HCV796 and PSI-352938, a cyclic monophosphate prodrug of 2′-alfa-F-2′-beta-C-methylguanosine. Double mutants in position C316, involved in resistance against compound HCV796, and Q309R, which is associated with a decreased response to IFN/ribavirin therapy, were also detected in two patients. In three out of four patients, the double mutant haplotype was also the dominant haplotype. As drug-resistant mutations can confer a decrease in viral fitness compared to wildtype viruses, it is surprising that they were observed as dominant haplotype in all patient. Possibly compensatory mutations may have evolved in these viruses to increase viral fitness. Unlike deep-sequencing platforms with very short read lengths, such as Illumina, our 454-sequencing approach provides the opportunity to look at the linkage of mutations and identification of double-resistant virus variants, provided that they are located in the same amplicon.

Several other mutations associated with resistance to response to IFN/ribavirin therapy were observed at much higher frequencies in DPS than with Sanger sequencing approaches^23–25^. The D244N, S326G, T329I, and D310N mutations were encountered as a minority variant in 10–12 patients (~60%). The Q309R mutation was encountered most frequently (in all patients) and even as a major haplotype in 4 patients, which is similar to previous observations^24^. However, when we analysed the presence of drug resistance populations in both compartments we noticed that the prevalence of the resistant variants was only somewhat higher in the liver as compared to plasma. Thus, the use of plasma is most likely sufficient to detect HCV quasispecies and drug-resistance associated variants. However, additional studies with large cohorts of paired samples, including analysis of other genome regions targeted by DAAs would be needed to reveal the clinical implications of the findings. Overall, our data thus provide insight into the HCV NS5B quasi-species population in liver and plasma in treatment-naïve patients obtained through state-of-the-art sensitive sequencing technologies.

## Materials and Methods

### Patients and samples

A total of eighteen patients chronically infected with HCV genotype 1b (HCV-1b) and naïve to any treatment were included. Plasma samples and liver biopsies were obtained and stored at −80 °C and RNA*late*r, respectively, until testing. The baseline clinical characteristics of patients are summarized in Supplementary Table S1. The amount of HCV RNA was determined by RT-PCR using Cobas Amplicor HCV Monitor version 2.0 (Roche Diagnostics, Branchburg, NJ) and HCV genotype was determined using INNO-LiPA HCV II (Innogenetics N.V., Ghent). All experimental protocols in this study was approved by the institutional ethics committees of Erasmus Medical Center, Rotterdam, the Netherlands. Informed consent was obtained from all subjects. All methods were performed in accordance with the relevant guidelines and regulations. Paired liver biopsies and plasma were only available from patients infected with genotype 1b in accordance with the approved study protocol for these specific patients.

### Viral RNA isolation, cDNA synthesis and PCR amplification

Viral RNA was extracted from 140 to 280 µl of plasma using the QIAamp viral RNA mini kit (Qiagen) and from liver biopsies (approximately 10 mg tissue was used) using RNeasy mini kit (Qiagen) and the RNA was eluted with 40 µl buffer according to the manufacturer’s instructions. To ensure a sufficient amount of viral copies for reliable detection of minor variants, the number of HCV RNA copies in the extracted RNA sample was determined by real-time quantitative polymerase chain reaction (qPCR). Ten microliter RNA was reverse transcribed with the Superscript III first-strand synthesis system (Invitrogen Corp) using random hexamers. The cDNA was used to amplify a 401 bp nucleotide genome fragment spanning amino acid positions 215 to 348 (nucleotides positions 8242 to 8642 according to GenBank accession no AJ238799) of the HCV NS5B polymerase gene with a nested PCR approach using the Hotstar Hifidelity Taq DNA polymerase (Qiagen). Both PCRs were carried out as follows: one initial denaturation step of 95 °C for 5 min, followed by 35 cycles of 95 °C for 30 s, 48 °C for 30 s, 72 °C for 40 s and a final extension of 72 °C for 10 min. The primers used were external sense primer Pri3, external antisense primer Pri4, and inner sense primer Pri1, inner antisense primer Pri2^44^. The inner sense and antisense primers were linked to DPS adapters A and B, respectively. To distinguish each sample in the multiplexed DPS, eight unique sequence tags were inserted between the adapter and the gene specific primer (Supplementary Table S2).

### 454 deep sequencing

PCR amplicons were purified from gel using the QIAquick gel extraction kit (Qiagen) and extracted DNA was again purified using Agencourt AMPure XP PCR purification system (Beckman Coulter). The quality and length of the amplicons were verified using Agilent 2100 bioanalyzer (Agilent Life Science, Santa Clara, California) and the concentration was quantified using Quant-iT PicoGreen dsDNA Reagent (Invitrogen) on a TECAN fluorometer (TECAN infinite F200). After quantification, amplicons were pooled in equimolar concentrations, followed by emulsion PCR (emPCR) and bead enrichment according to the 454 Titanium emPCR and enrichment bead recovery protocols (Roche), according to instructions of the manufacturer. The enriched beads were sequenced in both forward and reverse direction on the 454 Life Science platform (454 GS Junior, Roche Applied Science) according to the manufacturer’s instructions.

### Sanger sequencing

To analyse the deep sequencing data and to verify the sample authenticity, the NS5B polymerase gene from all plasma and liver tissue samples were PCR amplified as described above and sequenced directly on both strands using the BigDye Terminator version 3.1 Cycle sequencing kit on an ABI PRISM 3100 genetic analyser (Applied Biosystems). To exclude contamination between samples, the Sanger sequences were used to reconstruct a neighbour-joining phylogenetic tree with MEGA 5.05 software using the p-distance model with gamma distributed rate across sites (α = 0.5)^45^. Statistical support for internal branches in the tree was obtained by 1000 bootstrap replicates. The GenBank accession numbers of the sequences obtained in this study are KF730703-KF730738.

### Determination of errors introduced by reverse transcription, PCR and DPS

Errors caused by cDNA synthesis, PCR, or DPS are referred to as sequencing errors throughout the study. To quantify the frequency and nature of potential sequencing errors, we synthesized an HCV-1b NS5B polymerase plasmid (GenBank accession No: AJ238799) with T7 promoter (Eurogentech, Belgium). To discriminate between synthetic plasmid and natural HCV sequences, we introduced two mutations in the evolutionarily very well-conserved GDD (GHH) motif present in the active site for the HCV NS5B polymerase in the plasmid. To ensure this synthetic plasmid contained only one plasmid sequence, *E.coli* bacteria were transformed and a plasmid was isolated from a single bacterial clone. The sequence of the plasmid was confirmed by Sanger sequencing.

To assess the error rate of the polymerases in PCR in combination with DPS, the plasmid was quantified using Quant-iT PicoGreen dsDNA reagent, diluted to 10^6^ copies per ml (an input copy number similar to that of HCV RNA in samples), and amplified by conventional PCR. To measure the error rate of reverse transcriptase, RNA was synthesized from the plasmid using RiboMAX large scale RNA production system SP6 and T7 (Promega Corporation, Madison, WI, USA). Subsequently, template DNA was digested with DNase1 and absence of template plasmid in the RNA transcript was verified from serially diluted transcript using the nested PCR system as mentioned above. DNA-free RNA was reverse transcribed using the Superscript III reverse transcriptase and amplified by PCR using Hotstar Hifidelity Taq DNA polymerase (Qiagen). PCR amplified products were analyzed using the DPS protocol; the entire procedure from sample preparation to DPS was repeated three times in the two experiments using plasmid DNA or transcribed RNA.

### Deep sequencing data analysis

The deep sequencing reads were sorted into their sample of origin according to their unique sequence tag in the primers (Supplementary Table S2). The primer, tag, and adapter sequences were trimmed from the reads. If an average Phred quality score lower than 12 was encountered in a window of four bases across a read, the low-quality bases were removed and the read split at the respective position. Remaining reads were kept if they were longer than 200 bases. The trimming procedure was performed with scripts written in the Python programming language (Python 2.7.3) using Biopython tools (version 1.5.9)^46^.

Reads of each sample were aligned to the respective reference sequence with MOSAIK (version 1.3.88, https://code.google.com/p/mosaik-aligner/) as implemented in runMosaik.pl provided in ReadClean454 v1 (RC454)^47^. Aligned reads were corrected for homopolymer stretch polymorphisms (in homopolymers larger than 2N), for ‘carry forward and incomplete extension’ (CAFIE) errors^48^ and for insertion and deletions (InDels) causing frameshifts if they occurred in less than 25% of the reads with RC454. The corrected reads were passed to V-Phaser v1.0^49^, which combines information regarding covariation (“phasing”) in reads and an expectation maximization algorithm. Nucleotide and amino acid frequency tables were extracted with V-Profiler^47^. The amount of variation in the deep sequencing reads per nucleotide or translated amino acid position was derived from the frequency tables. Variations were considered as such if at least two high-quality reads exhibited the variant.

The average percentage of variation was determined for the entire amplicon of the control experiments. Furthermore, the variation per position encountered before read cleaning, which included the errors that were subsequently cleaned, was compared to the variation per position after read cleaning.

### Validation of read cleaning by deep sequencing analysis

To assess the validity of the read cleaning approach, two sets of reads with different 454 deep sequencing-specific error profiles were simulated from the plasmid sequence using Mason methodology^50^. First, 20000 reads were sampled with an error rate with the standard parameters of Mason-454, resulting in an average of 2.6 errors per reads. Second, 20000 reads with a higher error rate were simulated, with an average of 10.8 errors per read. The simulated reads were subjected to deep sequencing analysis as described above and the variation after read cleaning determined.

### Variant reconstruction

For haplotype reconstruction, the set of corrected reads was analyzed for redundancy. The amplified product was sequenced as a single fragment; therefore, the frequency of the haplotypes was directly estimated by counting the number of reads with an identical sequence. Each read was translated in all three possible reading frames. Translated reads that contained either an undetermined amino acid or a STOP codon were excluded as these do not represent genuine viruses. To ensure comparability among the samples and to exclude haplotypes generated by sequencing errors, only haplotypes that occurred at a frequency of at least 1% were selected for comparison. Alignments of the amino acid haplotype with a frequency of greater than or equal to 1% from liver and plasma of each patient was performed with Muscle v3.7 and distribution of haplotypes visualized using R statistical software version 2.13.1.

The diversity of the haplotypes was estimated using Simpson’s index of diversity 1-D^51^, which considers the number of haplotypes, the total number of sequences, and the proportion of each haplotype of the total number of sequences. This value ranges from 0–1 with 0 indicating low diversity and 1 indicating high diversity. Samples were grouped based on the average Simpson’s diversity index per patient (liver and plasma) and were considered to have low and high diversity when Simpson’s diversity index ≤0.5 or >0.5, respectively.

## Electronic supplementary material

Supplementary Tables
