# Supplementary material for: Identification of HCV Resistant Variants against Direct Acting Antivirals in Plasma and Liver of Treatment Naïve Patients
Source: Sci Rep. 2017 Jul 5;7:4688. doi: 10.1038/s41598-017-04931-y (PMC5498547; doi:10.1038/s41598-017-04931-y)
Supplement: Supplementary file 1 — Supplementary Tables [file 41598_2017_4931_MOESM1_ESM.pdf]

Supplementary Information

Identification of HCV Resistant Variants against Direct Acting Antivirals in Plasma and Liver of Treatment Naïve Patients

V. Stalin Raj[1], Gadissa Bedada Hundie[1], Anita C. Schürch[1], Saskia L. Smits[1,2] , Suzan D. Pas[1], Sophie Le Pogam[3], Harry L.A. Janssen[4,5], Rob J. de Knegt[4], Albert D.M.E. Osterhaus[6,7], Isabel Najera[3], Charles A. Boucher[1], Bart L. Haagmans[1*]

[1]Department of Viroscience, Erasmus Medical Center, Rotterdam, the Netherlands

[2]Viroclinics Biosciences BV, Rotterdam, the Netherlands

[3]Virology Discovery, Pharma Research Early Development Hoffmann La Roche, Nutley, NJ, USA

[4]Department of Gastroenterology and Hepatology, Erasmus Medical Center, Rotterdam, the Netherlands

[5]Division of Gastroenterology, University Health Network, Toronto, Canada

[6]Artemis One health, Utrecht, the Netherlands;

[7]Center for Infection Medicine and Zoonoses Research, University of Veterinary Medicine, Hannover, Germany

*Corresponding author. Bart L. Haagmans

E-mail: b.haagmans@erasmusmc.nl

Supplementary Tables

Table S1: Patient characteristics at baseline

Patient	Age (Years)	Sex[a]	Serum viral load (IU/ml)	ALT	Fibrosis
1	55	M	2.60×10^6	195	5
2	48	F	4.95×10^6	91	3
3	45	M	3.66×10^6	79	3
4	29	M	1.63×10^6	67	2
5	43	F	2.30×10^6	35	1
6	25	F	3.90×10^6	48	1
7	47	M	4.60×10^6	68	2
8	36	M	6.98×10^6	85	NA
9	54	M	1.71×10^6	107	4
10	54	M	4.20×10^6	59	3
11	37	F	7.81×10^5	31	3
12	45	F	1.65×10^6	43	0
13	53	M	1.84×10^6	19	1
14	39	M	5.13×10^6	86	5
15	27	M	1.65×10^6	61	2
16	21	M	4.81×10^5	65	1
17	31	M	2.23×10^6	41	NA
18	60	M	6.46×10^6	73	3

[a] M, male; F, female**;** NA, not available

Table S2: DPS primer sequence

No	Primer name	Adopter	Taq	MID	Genespecific primer
1	Pr1FNS5bMID1	5'-CGTATCGCCTCCCTCGCGCCA	TCAG	ACGAGTGCGT	TGGGGATCCCGTATGATACCCGCTGCTTTGA-3'
2	Pr2RNS5bMID2	5'-CTATGCGCCTTGCCAGCCCGC	TCAG	ACGCTCGACA	GGCGGAATTCCTGGTCATAGCCTCCGTGAA-3'
3	Pr2RNS5bMID3	5'-CTATGCGCCTTGCCAGCCCGC	TCAG	AGACGCACTC	GGCGGAATTCCTGGTCATAGCCTCCGTGAA-3'
4	Pr2RNS5bMID4	5'-CTATGCGCCTTGCCAGCCCGC	TCAG	AGCACTGTAG	GGCGGAATTCCTGGTCATAGCCTCCGTGAA-3'
5	Pr2RNS5bMID5	5'-CTATGCGCCTTGCCAGCCCGC	TCAG	ATCAGACACG	GGCGGAATTCCTGGTCATAGCCTCCGTGAA-3'
6	Pr2RNS5bMID6	5'-CTATGCGCCTTGCCAGCCCGC	TCAG	ATATCGCGAG	GGCGGAATTCCTGGTCATAGCCTCCGTGAA-3'
7	Pr2RNS5bMID7	5'-CTATGCGCCTTGCCAGCCCGC	TCAG	CGTGTCTCTA	GGCGGAATTCCTGGTCATAGCCTCCGTGAA-3'
8	Pr2RNS5bMID8	5'-CTATGCGCCTTGCCAGCCCGC	TCAG	CTCGCGTGTC	GGCGGAATTCCTGGTCATAGCCTCCGTGAA-3'

MID- multiples identifier

Table S3: Amount of HCV reads, haplotypes, and Simpson's diversity index per liver (L) or plasma (P) sample of each patient.

Patient	No of reads	No of "cleaned" reads	No reads of haplotypes with frequencies of more than 1%	No haplotypes	Simpson's Diversity Index
1L	16049	15650	13588	4	0.14
1P	17913	17540	15725	2	0.08
2L	18885	18411	16381	4	0.23
2P	18361	18010	16240	6	0.21
3L	14094	13672	8185	11	0.77
3P	27949	27102	23350	2	0.06
4L	27468	26241	21966	6	0.48
4P	29496	28706	23879	5	0.48
5L	29960	28893	23641	6	0.47
5P	31467	30685	24433	5	0.45
6L	25874	24743	19848	2	0.5
6P	26824	25742	21612	4	0.37
7L	29932	27883	22646	5	0.29
7P	27569	26828	21128	4	0.41
8L	24848	24111	21144	3	0.06
8P	21393	20198	16800	5	0.24
9L	17643	16750	10546	14	0.89
9P	21213	20267	15883	6	0.37
10L	13760	12948	9933	7	0.82
10P	26634	25865	21036	4	0.58
11L	16836	16013	13191	4	0.45
11P	24273	23343	19020	7	0.58
12L	14365	13498	12056	3	0.28
12P	22374	21430	18806	6	0.3
13L	17245	16327	14248	4	0.15
13P	23966	22655	19090	5	0.33
14L	33251	31536	24757	7	0.61
14P	36010	34662	28486	7	0.64
15L	28058	27543	24066	5	0.49
15P	37164	36292	31941	4	0.48
16L	32415	30081	22151	4	0.24
16P	22707	22075	19664	2	0.02
17L	28402	27409	21067	10	0.72
17P	25854	24885	21212	5	0.56
18L	8605	8400	7089	5	0.56
18P	23761	23209	19571	3	0.26

Table S4: Detection of HCV-NS5B resistant variants by DPS (% of total cleaned reads).

Patient	S282T	C316N	C316H	C316Y	V321I	D244N	Q309R	D310N	S326G	T329I	A333E
cutoff	0	0.2	0.2	0.2	0	0	0	0	0	0	0.4
1L	0	99.9	0	0	0	0	0.23	0.02	0	0	0
1P	0	99.92	0	0	0	0	0	0	0	0	0
2L	0	0	0	0	0	0	0	0	0	0	0
2P	0	4.38	0	0	0.03	0	0.54	0	0	0	0
3L	0	65.66	3.12	5.63	0	0.02	5.82	0	0	0.02	0
3P	0	99.81	0	0	0	0	0.18	0.03	0	0.07	0
4L	0	99.93	0	0	0.02	0	0.11	0	0	0	0
4P	0	99.88	0	0	0	0.02	0.19	0.04	0.03	0	0
5L	0	20.77	0	0	0	0	0.25	0.22	0.03	0.04	0
5P	0	0	0	0	0.04	0.05	0.21	0.08	0.06	0.07	0
6L	0	99.92	0	0	0.02	0	0.35	0.88	0	0	0
6P	0	99.83	0	0	0	0	0.21	0	0	0	0
7L	0	0.28	0	0	0.02	0.11	0.51	0.02	0.02	0.08	0
7P	0	0	0	0	0.01	0.04	0.62	0.03	0.06	0.09	0
8L	0	0.34	0	99.47	0.03	0.07	99.53	0.07	0.02	0.14	0
8P	0	13.66	0.8	85.26	0	0.04	85.28	0.03	0.08	0.07	0
9L	0	11.55	43.91	1.39	0.02	0.01	2.42	0.2	0.17	0.2	0
9P	0	13.27	0	0	0	0.13	0.48	0.02	0.03	0.16	0
10L	0	73.55	0.64	0	0	0	0.9	0.1	0	0.12	0
10P	0	99.56	0	0	0	0.08	0.4	0	0.05	0.07	0
11L	0	0	0	0	0.03	0	0.68	0	0.07	0.03	0
11P	0	0	0	0	0	0	0.42	0.03	0.06	0.11	0
12L	0	0.3	0.65	0	0	0	0.16	0	0	0	0
12P	0	7.31	0	0	0	0.04	0.28	0	0.03	0	0
13L	0	0	1.7	0	0	0	94.17	0	0	0.01	0
13P	0	0	0	0	0	0	83.41	0	0.11	0.17	0
14L	0	0	97.41	0	0	0	2.53	0.09	0.28	0.05	0
14P	0	0	99.73	0	0.02	0.01	0.88	0.02	0	0.04	0
15L	0	99.93	0	0	0.03	0.09	72.61	0.02	0	0.85	0
15P	0	99.91	0	0	0	0.06	71.42	0	0	0.8	0
16L	0	99.84	0	0	0	0	0.25	0	0.05	0	0
16P	0	97.78	0	0	0	0	0.18	0	0	0	0
17L	0	2.9	0	0	0	0	94.69	0	0.01	0.03	0
17P	0	0.55	0	0	0	0	98.82	0	0	0.08	0
18L	0	0	0	0	0	0	0.23	0	0	0	0
18P	0	0	0	0	0	0	0.39	0	0	0	0

L, liver; P, plasma. Cut-off, position specific error rate determined in control experiments.
